# A comparison of the predictive accuracy of three screening models for pulmonary arterial hypertension in systemic sclerosis

**DOI:** 10.1186/s13075-015-0517-5

**Published:** 2015-01-18

**Authors:** Yanjie Hao, Vivek Thakkar, Wendy Stevens, Kathleen Morrisroe, David Prior, Candice Rabusa, Peter Youssef, Eli Gabbay, Janet Roddy, Jennifer Walker, Jane Zochling, Joanne Sahhar, Peter Nash, Susan Lester, Maureen Rischmueller, Susanna M Proudman, Mandana Nikpour

**Affiliations:** Department of Rheumatology, St Vincent’s Hospital Melbourne, 41 Victoria Parade, Fitzroy, VIC 3065 Australia; Department of Rheumatology and Clinical Immunology, Peking University First Hospital, 8 Xishiku Street, Beijing, China; Department of Rheumatology, Liverpool Hospital, Elizabeth Street, Liverpool, NSW 2170 Australia; School of Medicine, University of Western Sydney, Locked bag 1797, Penrith, NSW 2751 Australia; Department of Cardiology, St Vincent’s Hospital Melbourne, 41 Victoria Parade, Fitzroy, VIC 3065 Australia; Institute of Rheumatology and Orthopaedics, Royal Prince Alfred Hospital, Queen Elizabeth II building, Missendon Road, Camperdown, NSW 2050 Australia; The University of Notre Dame, 19 Mouat Street, Fremantle, WA 6959 Australia; Department of Rheumatology, Royal Perth Hospital, Wellington Street, GPO Box X2213, Perth, WA 6001 Australia; Department of Rheumatology, Flinders Medical Centre, Flinders Drive, Bedford Park, SA 5042 Australia; Department of Rheumatology, The Menzies Institute Tasmania, Private Bag 23, Hobart, TAS 7001 Australia; Department of Rheumatology, Monash Medical Centre, 246 Clayton Road, Clayton, VIC 3168 Australia; Rheumatology Research Unit, Department of Medicine, University of Queensland, PO Box 368, Maroochydore, QLD 4558 Australia; Rheumatology Department, The Queen Elizabeth Hospital, 28 Woodville Road, Woodville South, SA 5011 Australia; Department of Rheumatology, Royal Adelaide Hospital, North Terrace, SA 5000 Australia; Discipline of Medicine, University of Adelaide, North Terrace, SA 5000 Australia; Department of Medicine at St Vincent’s Hospital Melbourne, The University of Melbourne, 41 Victoria Parade, Fitzroy, VIC 3065 Australia

## Abstract

**Introduction:**

There is evidence that early screening for pulmonary arterial hypertension (PAH) in systemic sclerosis (SSc) improves outcomes. We compared the predictive accuracy of two recently published screening algorithms (DETECT 2013 and Australian Scleroderma Interest Group (ASIG) 2012) for SSc-associated PAH (SSc-PAH) with the commonly used European Society of Cardiology/European Respiratory Society (ESC/ERS 2009) guidelines.

**Methods:**

We included 73 consecutive SSc patients with suspected PAH undergoing right heart catheterization (RHC). The three screening models were applied to each patient. For each model, contingency table analysis was used to determine sensitivity, specificity, and positive (PPV) and negative (NPV) predictive values for PAH. These properties were also evaluated in an ‘alternate scenario analysis’ in which the prevalence of PAH was set at 10%.

**Results:**

RHC revealed PAH in 27 (36.9%) patients. DETECT and ASIG algorithms performed equally in predicting PAH with sensitivity and NPV of 100%. The ESC/ERS guidelines had sensitivity of 96.3% and NPV of only 91%, missing one case of PAH; these guidelines could not be applied to three patients who had absent tricuspid regurgitant (TR) jet. The ASIG algorithm had the highest specificity (54.5%). With PAH prevalence set at 10%, the NPV of the models was unchanged, but the PPV dropped to less than 20%.

**Conclusions:**

In this cohort, the DETECT and ASIG algorithms out-perform the ESC/ERS guidelines, detecting all patients with PAH. The ESC/ERS guidelines have limitations in the absence of a TR jet. Ultimately, the choice of SSc-PAH screening algorithm will also depend on cost and ease of application.

**Electronic supplementary material:**

The online version of this article (doi:10.1186/s13075-015-0517-5) contains supplementary material, which is available to authorized users.

## Introduction

Systemic sclerosis (SSc) is a multisystem connective tissue disease characterized by vasculopathy and fibrosis. Pulmonary arterial hypertension (PAH) is one of the most severe organ complications and a leading cause of death in SSc. Despite advanced PAH therapies, the 3-year survival of SSc-associated PAH (SSc-PAH) is around 50% [1]. Recent evidence indicates that the earlier treatment is started in the course of disease, the better the prognosis [2-4]. Therefore, early detection of PAH has become an important consideration in the optimal management of patients with SSc.

The most commonly used pulmonary hypertension screening guidelines from the European Society of Cardiology/European Respiratory Society (ESC/ERS) are based on symptoms and transthoracic echocardiography (TTE) [5]. But there are limitations in symptom- and TTE-based algorithms. In the early stages, the symptoms of PAH are usually very mild and non-specific, making it difficult to identify patients who are developing PAH. In patients with SSc, coexisting organ involvement such as interstitial lung disease (ILD) makes the diagnosis of PAH even more challenging. In addition, the most widely used echocardiographic parameter, tricuspid regurgitant jet velocity (TRV), is not present in all patients. In fact, TRV cannot be obtained in 20% to 39% of patients, potentially decreasing the sensitivity of TTE-based algorithms [6,7]. Another consideration is the cost-effectiveness of TTE-based screening.

These limitations of current screening algorithms emphasize the need for alternative approaches to improve the selection of patients for referral for right heart catheterization (RHC), the ‘gold standard’ test for the diagnosis of PAH. Emerging screening algorithms incorporate pulmonary function tests (PFTs) and biomarkers such as N-terminal pro-B type natriuretic peptide (NT-proBNP) [8-12]. In 2012, the Australian Scleroderma Interest Group (ASIG) developed a screening algorithm for SSc-PAH by using serum NT-proBNP level and PFT [11]; this was found to have similar sensitivity and higher specificity and positive (PPV) and negative (NPV) predictive value in comparison with the ESC/ERS guidelines [13].

The DETECT (Evidence-Based Detection of Pulmonary Arterial Hypertension in Systemic Sclerosis) study investigators recently developed a new detection algorithm for PAH in patients with SSc [14]. This study included 644 patients with diffusing capacity for carbon monoxide (DLCO) of less than 60% predicted, from 18 countries in North America, Europe, and Asia. The algorithm combined eight variables—telangiectasia, anti-centromere antibody (ACA), NT-proBNP, serum urate, forced vital capacity (FVC) percentage predicted/DLCO percentage predicted (FVC/DLCO) on PFT, right axis deviation on electrocardiogram (ECG), right atrium (RA) area, and TRV on TTE—and established a two-step decision tree, which improved the sensitivity of screening for SSc-PAH from 71% to 96% in comparison with the ESC/ERS guidelines. However, to date, the performance of the DETECT algorithm has not been evaluated among patients who were not included in the derivation study. Therefore, the aims of this study were to validate the predictive accuracy of the DETECT algorithm in Australian patients with SSc and to compare the performances of DETECT and ASIG algorithms with the ESC/ERS guidelines.

## Methods

### Patients

Patients included in this analysis were from the Australian Scleroderma Cohort Study (ASCS). The ASCS is a multi-center study of risk and prognostic factors for cardiopulmonary outcomes in SSc. All patients fulfil either American College of Rheumatology or Leroy and Medsger criteria for SSc [15,16]. The ASCS has been approved by the human research ethics committees of the 13 participating Australian centers (St Vincent’s Hospital Melbourne, Royal Perth Hospital, Royal Adelaide Hospital, The Queen Elizabeth Hospital, Sunshine Coast Rheumatology, Prince Charles Hospital, John Hunter Hospital, Royal Prince Alfred Hospital, Royal North Shore Hospital, St George Hospital, Canberra Rheumatology, Monash Medical Centre, and The Menzies Research Institute Tasmania). All patients provide written informed consent at recruitment.

### Inclusion and exclusion criteria

We included consecutive adult (≥18 years) SSc patients from the ASCS between December 2007 and December 2012, who were considered to be at high risk for PAH according to the ASCS screening guidelines and had undergone RHC. The existing Australian screening guidelines require that all patients undergo an annual clinical assessment, TTE and PFTs. Any patient identified as having possible PAH—that is, systolic pulmonary arterial pressure (sPAP_TTE_) of at least 40 mm Hg, and/or DLCO less than 50% predicted with FVC of more than 85% predicted, without adequate explanation on high-resolution computed tomography (HRCT) lung or ventilation-perfusion (V/Q) scanning or both—undergoes RHC.

In addition to undergoing RHC, all patients have serum collected for NT-proBNP measurement within 1 month of their RHC and, in cases of PAH, prior to the commencement of advanced pulmonary vasodilator therapy.

In keeping with the exclusion criteria of the DETECT study [14], patients were excluded if they had pulmonary hypertension (PH) confirmed by RHC prior to enrolment, were receiving advanced PH therapy, had an FVC of less than 40% of predicted or renal insufficiency, or were pregnant. As per the DETECT algorithm, patients with more than one missing variable among eight variables were also excluded.

For validating the performance of the ESC/ERS guidelines, patients without detectable TRV were excluded. For validating the performance of the ASIG algorithm, patients involved in the derivation study [13] for this algorithm were excluded.

### Definitions

Based on the current World Health Organization (WHO) classification, in this study, patients were classified as non-PH or WHO group 1 PH (PAH), WHO group 2 PH (left heart disease-associated PH), or WHO group 3 PH (lung disease/hypoxia-associated PH). PH was defined as a mean pulmonary artery pressure (mPAP) of at least 25 mm Hg on RHC at rest; therefore, non-PH was defined as mPAP of less than 25 mm Hg. PAH was defined as mPAP of at least 25 mm Hg at rest and pulmonary capillary wedge pressure (PCWP) of not more than 15 mm Hg with no more than mild ILD on HRCT and an FVC of more than 60% predicted. WHO group 2 PH was defined as mPAP of at least 25 mm Hg at rest and PCWP of more than 15 mm Hg. WHO group 3 PH was defined as mPAP of at least 25 mm Hg at rest and PCWP of not more than 15 mm Hg with FVC of less than 70% predicted plus moderate or severe ILD on HRCT [5,14]. Patients with PH underwent CT pulmonary angiography or V/Q imaging to exclude chronic thrombo-embolic PH (WHO group 4 PH).

### Screening algorithms

**Figure 1:**
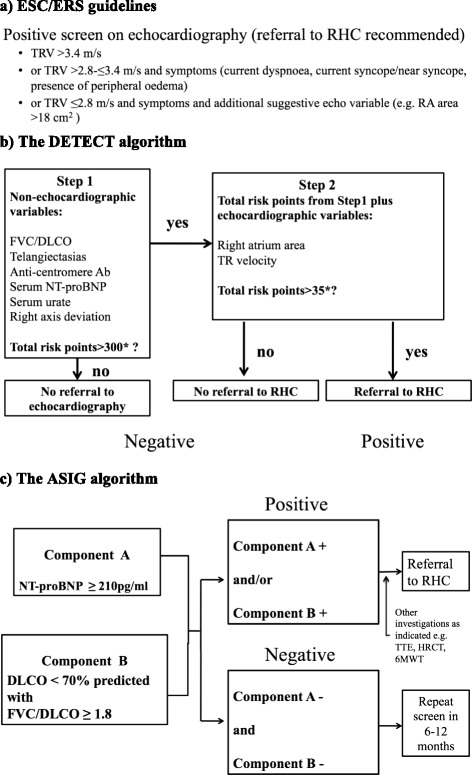
**Summary of screening algorithms.** Ab, antibody; ASIG, Australian Scleroderma Interest Group; DLCO, diffusing capacity for carbon monoxide; ESC/ERS, European Society of Cardiology/European Respiratory Society; FVC, forced vital capacity (percentage predicted); HRCT, high-resolution computed tomography (chest); PAH, pulmonary arterial hypertension; PH, pulmonary hypertension; RA, right atrium; RHC, right heart catheterization; SSc, systemic sclerosis; TR, tricuspid regurgitation; TRV, tricuspid regurgitant velocity; TTE, transthoracic echocardiography; WHO, World Health Organization.

As described above, the DETECT screening algorithm (Figure 1) includes eight variables and a two-step decision tree. At ‘step 1’, risk points for each of the six non-echocardiographic variables (FVC/DLCO, telangiectasia, anti-centromere antibody (ACA), NT-proBNP, urate, and ECG right axis deviation) are calculated by using nomograms as presented in the article and verified by using a customized calculator sourced from a dedicated website [17]. These are added together to obtain the ‘total step 1 risk points’. If the ‘total risk points from step 1’ are more than 300, the patient is referred for TTE and entered into ‘step 2’. At ‘step 2’, the risk points for the two echocardiographic variables (RA area and TRV) are calculated and added to obtain a total score for step 2. If the ‘total risk points from step 2’ are more than 35, the patient is referred to RHC. Overall, this means that a positive screen for RHC referral in DETECT is a score of 300+ in ‘step 1’ together with a score of 35 or more in ‘step 2’ [14].

The ASIG screening algorithm (Figure 1) is composed of two components: PFT (component A) and serum NT-proBNP level (component B). Component A is present if DLCO is less than 70% predicted with an FVC/DLCO of at least 1.8, and component B is present if NT-proBNP is at least 210 pg/mL. In this model, the screen is ‘positive’ if component A, component B, or both components A and B are present, and a screen is ‘negative’ if both component A and component B are absent. All patients with a positive screen move on to TTE together with further tests such as HRCT, V/Q, and 6-minute walk test (6MWT) as clinically indicated, mainly in order to exclude the other contributing factors for PH (left heart dysfunction, ILD, and pulmonary embolism). If no alternative explanation is found for a positive screen, patients undergo confirmatory RHC testing, regardless of sPAP at echocardiography [11].

A positive screen for RHC referral in the ESC/ERS guidelines (Figure 1) is TRV of more than 3.4 m/s or 2.8 < TRV ≤ 3.4 m/s with symptoms (defined as at least one of the following parameters: current dyspnea, current syncope/near syncope, presence of peripheral edema) or TRV of not more than 2.8 m/s and above symptoms, together with an additional suggestive echocardiographic variable (defined as RA area of more than 18 cm^2^) [5].

In summary, TTE is a component of both the ESR/ERS guidelines and the DETECT algorithm. In the former, it is the sole investigation component; in the later, TTE forms ‘step 2’ of the algorithm and is required if a patient’s points from step 1 are greater than 300. TTE is not mandated in the ASIG algorithm, although it is recommended in patients who screen positive, in order to obtain more information about cardiac valves and myocardial function. Step 1 of the DETECT algorithm requires six variables: three measured in blood tests (including NT-proBNP), one determined by ECG (right axis deviation), one determined by pulmonary function tests (FVC/DLCO ratio), and another determined by physical examination (telangiectasia). In contrast, the ASIG algorithm is composed of two tests—a single blood test for NT-proBNP and pulmonary function tests—to determine DLCO percentage predicted and the FVC/DLCO ratio.

### Data collection

Data such as demographic and clinical variables and cardiac and pulmonary assessments were obtained from the ASCS database. All physical examination and investigation data were collected within 1 month of the first RHC, before starting advanced PAH therapy. TTE was performed according to standardized procedures only at tertiary centers. Pulmonary involvement was assessed by PFTs or HRCT or both. All DLCO values were reported as percentage predicted values and corrected for hemoglobin [18]. All patients had serum collected for NT-proBNP measurement within 1 month of their RHC and, in cases of PAH, prior to the commencement of advanced PAH therapy.

### Statistical analysis

Data are presented as mean ± standard deviation for continuous variables and as number (percentage or proportion) for categorical variables. Normally distributed continuous variables were compared by using the Student *t* test with unequal variances, whereas continuous non-parametric variables were compared by using Kruskall-Wallis and Mann-Whitney *U* tests. The differences in frequency were determined by using chi-square and Fisher’s exact tests. The predictive accuracy of the algorithms is presented as sensitivity, specificity, PPV and NPV, with 95% confidence intervals (CIs). An ‘alternate case scenario’ analysis (detailed in Additional file 1) was also performed by assuming a prevalence for PAH of 10%, the commonly accepted frequency of PAH in SSc. A two-tailed *P* value of not more than 0.05 was considered statistically significant. All statistical analyses were performed by using STATA 13.0 (StataCorp LP, College Station, TX, USA).

## Results

### Patient characteristics

Of 79 consecutive SSc patients with suspected PAH undergoing RHC who fulfilled the inclusion criteria, RHC revealed PH in 45 (57.0%) patients and PAH (WHO group 1) in 29 (36.7%) patients. Among them, six patients were excluded for the following reasons: three because of FVC of less than 40% predicted and three because they had more than one missing variable in the DETECT algorithm. Of the remaining 73 patients, in the DETECT algorithm analysis, four patients were missing RA area only, two patients were missing urate level only, and 12 patients were missing ECG data. There were no missing NT-proBNP data.

**Figure 2:**
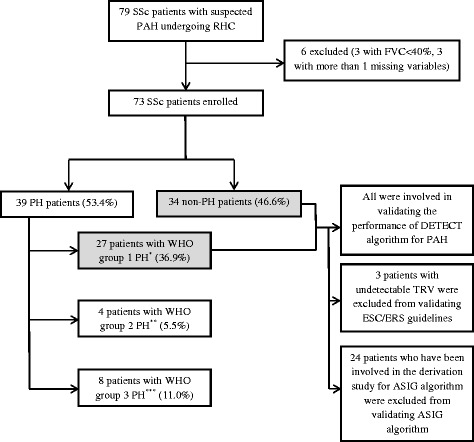
**Study cohort.** *World Health Organization (WHO) group 1 pulmonary hypertension (PH) means pulmonary arterial hypertension (PAH). **WHO group 2 PH means left heart disease-associated PH. This group was excluded for validating the performance of all models for PAH but included in validating the performance for PH. ***WHO group 3 PH means lung disease/hypoxia-associated PH. This group was excluded for validating the performance of all models for PAH but included in validating the performance for precapillary PH and PH. ASIG, Australian Scleroderma Interest Group; ESC/ERS, European Society of Cardiology/European Respiratory Society; FVC, forced vital capacity (percentage predicted); RHC, right heart catheterization; SSc, systemic sclerosis; TRV, tricuspid regurgitant velocity.

Among the 73 patients, PH was confirmed in 39 (53.4%) patients: 27 (36.9%) with PAH, 4 (5.5%) in the WHO group 2 PH, and 8 (11.0%) in the WHO group 3 PH. Finally, 61 patients (27 PAH patients versus 34 non-PH patients) were included in the analyses of the performance of the algorithms for PAH. In three patients, TRV was undetectable, and in these cases the ESC/ERS guidelines could not be applied. Twenty-four patients had been included in the derivation study [13] for the ASIG algorithm and were not used in the analysis of the ASIG algorithm. WHO group 2 (n = 4) PH patients were included in further performance analyses for PH. WHO group 3 (n = 8) PH patients were included in further performance analyses for precapillary PH and PH (Figure 2).

### Comparison between pulmonary arterial hypertension and non-pulmonary hypertension groups

Compared with non-PH patients, PAH patients were older at the time of diagnosis of PAH, even though their age at the onset of SSc was very similar. Unlike the sex distribution of the DETECT study, in this cohort, patients with PAH were more likely to be female than patients without PH (93% versus 76%, *P* = 0.027). Similar to previous reports, PAH patients were more likely to have the limited subtype of SSc, to be ACA-positive, and to have telangiectasia than non-PH patients. As expected, patients with PAH had a shorter 6-minute walk distance (6MWD), lower DLCO level, higher NT-proBNP level, larger RA area, and higher TRV than the non-PH group. The FVC percentage predicted values were similar between the two groups, but FVC/DLCO ratio was significantly higher in the PAH group than in the non-PH group. We did not find any difference in the frequency of ECG right axis deviation between the two groups, but significantly higher serum urate levels were found in the PAH group (Tables 1 and 2).

**Table 1:** **Comparison of the clinical characteristics of pulmonary arterial hypertension and non-pulmonary hypertension groups**

**Characteristics at the time of screening**	**PAH**	**Non-PH**	***P*** **value**
	**(n = 27)**	**(n = 34)**	
	**Mean ± SD or n (%)**	**Mean ± SD or n (%)**	
**Age at disease onset, years** ^**a**^	52.9 ± 14.7	48.6 ± 13.2	0.256
**Age at study, years**	66.8 ± 8.3	61.2 ± 11.9	0.033
**Disease duration at study, years**	14.9 ± 12.6	12.8 ± 10.4	0.566
	13.0 (3.2-21.9)^b^	10.3 (4.4-18.2)^b^	
**Female**	25 (93)	16 (76)	0.027
**SSc subtypes**			
**Limited**	23 (85)	27 (79)	0.560
**Diffuse**	4 (15)	7 (21)	
**Telangiectasia**	24 (89)	23 (68)	0.049
**Antibodies**			
**ANA**	24 (89)	27 (90)	0.617
**Anti-Scl70**	1 (4)	3 (10)	0.373
**Anti-cent**	17 (63)	11 (32)	0.017
**NT-proBNP, pg/mL**	1,619.6 ± 2,063.6	401.3 ± 689.4	<0.0001
	841 (531.3-2,022.1)^b^	143.6 (83.3-414.2)^b^	
**Serum urate, mg/100 mL**	6.7 ± 2.2	5.6 ± 1.8	0.038
**6MWD, m**	310.4 ± 115.8	411.0 ± 114.2	0.003

^a^Disease onset defined as the date of first non-Raynaud’s symptom. ^b^Median (interquartile range). 6MWD, six-minute walk distance; ANA, anti-nuclear antibody; anti-cent, anti-centromere antibody; anti-Scl70, anti-topoisomerase-1 antibody; NT-proBNP, N-terminal pro-B type natriuretic peptide; PAH, pulmonary arterial hypertension; PH, pulmonary hypertension; SD, standard deviation; SSc, systemic sclerosis.

**Table 2:** **Comparison of cardiac and pulmonary investigation parameters in pulmonary arterial hypertension and non-pulmonary hypertension groups**

**Investigations**	**PAH**	**Non-PH**	***P*** **value**
	**(n = 27)**	**(n = 34)**	
	**Mean ± SD or n (%)**	**Mean ± SD or n (%)**	
**TTE parameters**			
**RA area, cm** ^**2**^	20.8 ± 6.6	17.6 ± 4.2	0.038
**TRV, m/s**	3.4 ± 0.6	2.7 ± 0.9	0.0002
**sPAP, mm Hg**	57.7 ± 19.6	41.6 ± 10.6	0.0004
**RAD** ^**a**^ **on ECG**	3 (13)	3 (12)	0.073
**PFT results**			
**FVC, % pred**	91.6 ± 15.1	90.9 ± 25.6	0.900
**DLCO** ^**b**^ **, % pred**	47.9 ± 11.7	60.6 ± 14.9	0.0008
**FVC/DLCO**	2.0 ± 0.5	1.6 ± 0.4	0.0003
**RHC results**			
**mPAP, mm Hg**	36.4 ± 10.1	19.4 ± 3.4	<0.0001
**mRAP, mm Hg**	9.7 ± 3.9	5.5 ± 2.9	0.0001
**PVR, Wood units**	5.7 ± 3.3	1.9 ± 0.9	<0.0001

^a^Right axis deviation (RAD) defined as QRS axis of at least 90°. ^b^Diffusing capacity for carbon monoxide (DLCO) values are reported as percentage predicted values, corrected for hemoglobin. ECG, electrocardiogram; FVC, forced vital capacity (percentage predicted); mPAP, mean pulmonary artery pressure; mRAP, mean right atrial pressure; PAH, pulmonary arterial hypertension; PFT, pulmonary function test; PH, pulmonary hypertension; pred, predictive value; PVR, pulmonary vascular resistance; RA, right atrium; RHC, right heart catheterization; SD, standard deviation; sPAP, systolic pulmonary artery; TRV, tricuspid regurgitant velocity; TTE, transthoracic echocardiography.

### Performance of DETECT, ESC/ERS, and ASIG screening models for pulmonary arterial hypertension

The sensitivity, specificity, PPV, and NPV of the DETECT algorithm for PAH in this cohort were 100%, 35.3%, 55.1%, and 100%, respectively. At the end of ‘step 1’, the median (interquartile range) score of all 61 patients was 330.4 (315.1 to 340.5), and 53 patients would be referred for TTE and proceed to ‘step 2’, so the referral rate for TTE was 87%. In ‘step 2’, the median (interquartile range) score of 53 included patients was 48.9 (37.2 to 55.3). Finally, 49 patients attained a score that would result in referral for RHC, and the final referral rate was 80%. No PAH patient was missed by the algorithm, but 22 patients who would have been referred for RHC were found to have non-PH on RHC. When the DETECT algorithm was used, 45% of RHCs did not confirm a diagnosis of PAH.

Compared with the DETECT algorithm, the ASIG algorithm performed equally well in sensitivity (100%) and NPV (100%), a little better in PPV (60%), and moderately better in specificity (54.5%). The referral rate for RHC was 68%, and the proportion of RHCs that did not confirm a diagnosis of PAH was 40%.

Compared with the other two algorithms, the ESC/ERS guidelines had the lowest sensitivity, specificity, and NPV, which were 96.3%, 32.3%, and 90.9%, respectively, and similar PPV of 55.3%. One PAH patient was missed and 47 patients would have been referred for RHC with a referral rate of 81%. The proportion of RHCs that did not confirm a diagnosis of PAH was 45%. The ‘missed’ PAH patient had a mildly increased pulmonary pressure with mPAP of 26 mm Hg on RHC. Because of very mild TR jet with TRV of 2.2 m/s and normal RA area of 14 cm^2^, this patient was missed by the ESC/ERS guidelines. This patient had telangiectasia, negative ACA antibody, normal NT-proBNP level of 73.7 pg/mL, no right axis deviation on ECG, well-preserved WHO functional class (WHO FC) of 2, and 6MWD of 467 m, but PFT showed a very low DLCO predicted value of 44% and high FVC/DLCO ratio of 2.27, with a normal FVC percentage predicted value of 100%. The performance characteristics of the three models for PAH are presented in Table 3.

**Table 3:** **Comparison of the performance of DETECT versus ESC/ERS versus ASIG screening models for pulmonary arterial hypertension in patients with systemic sclerosis**

				**PAH prevalence set at 10%** ^**a**^		
	**DETECT**	**ESC/ERS**	**ASIG**	**DETECT**	**ESC/ERS**	**ASIG**
	**n = 61**	**n = 58**	**n = 37**	**n = 61**	**n = 58**	**n = 37**
**Positive** ^**b**^	49 (80.3)	48 (82.8)	25 (67.6)			
**Negative** ^**b**^	12 (19.7)	10 (17.2)	12 (32.4)			
**True PAH on RHC** ^**c**^	27 (44.3)	27 (46.55)	15 (40.54)			
**Sensitivity**	100%	96.3%	100%	100%	96.3%	100%
**(95% CI)**	(87.2-100)	(81.0-99.9)	(78.2-100)	(54.1-100)	(54.1-100)	(39.8-100)
**Specificity**	35.3%	32.3%	54.5%	35.3%	32.3%	54.5%
**(95% CI)**	(19.7-53.5)	(16.7-51.4)	(32.2-75.6)	(23.8-50.4)	(15.6-41.0)	(33.5-69.2)
**PPV**	55.1%	55.3%	60%	14.7%	13.6%	19.6%
**(95% CI)**	(40.2-69.3)	(40.1-69.8)	(38.7-78.8)	(5.6-29.2)	(5.2-27.4)	(5.7-43.7)
**NPV**	100%	90.9%	100%	100%	98.7%	100%
**(95% CI)**	(63.1-100)	(58.7-99.8)	(73.5-100)	(83.2-100)	(76.8-100)	(80.5-100)

^a^Refer to Additional file 1. ^b^Positive or negative number screened by each of the algorithms. Values are presented as number (percentage). ^c^True pulmonary arterial hypertension (PAH) number confirmed by right heart catheterization (RHC). Values are presented as number (percentage). ASIG, Australian Scleroderma Interest Group; CI, confidence interval; ESC/ERS, European Society of Cardiology/European Respiratory Society; NPV, negative predictive value; PPV, positive predictive value.

### Alternate case scenario analysis for pulmonary arterial hypertension

The prevalence of PAH in this cohort was 37%, which was higher than the expected prevalence of 10% according to the commonly reported data in the literature [19]. This was because these patients had been selected on the basis of the existing Australian guidelines summarized above. So, assuming a prevalence of PAH of 10%, we performed an alternate case scenario analysis, using the point estimates of sensitivity and specificity determined above. The NPV of the models was unchanged, but as would be expected, the PPV of all three models dropped significantly below 20% (Table 3 and Additional file 1).

### Differences in immunosuppressive therapy in screen-positive and screen-negative patients

Use of immunosuppressive therapy (defined as any exposure irrespective of dose or duration) in the 12 months immediately preceding the date of screening by patients who screened positive and patients who screened negative according to each algorithm was compared. There were no statistically significant differences in the use of cyclophosphamide, methotrexate, or mycophenolate for each algorithm, nor for azathioprine or corticosteroids for the ESC/ERS and ASIG algorithms. Interestingly, azathioprine and corticosteroids were used by more patients who screened positive than patients who screened negative using the DETECT algorithm (1/49 versus 3/12, *P* <0.0001 for azathioprine; 6/49 versus 7/12, *P* <0.0001 for corticosteroids), suggesting that these drugs may be associated with one or more of the parameters in the DETECT algorithm such as raised serum urate, although the small number of patients on these medications prevents us from drawing definitive conclusions in this regard.

### Performance of DETECT, ESC/ERS, and ASIG screening models for precapillary pulmonary hypertension and pulmonary hypertension

The performance of these screening models for precapillary PH (WHO group 1 and 3 PH) and for all types of PH patients (WHO group 1, 2, and 3 PH) was also evaluated. We did this because it could be argued that these ‘screening’ algorithms need to detect patients at high risk of PH, regardless of etiology, which then can be further elucidated with ‘diagnostic’ testing. For precapillary PH, the ASIG algorithm performed best with respect to sensitivity (100%) and NPV (100%), without missing any true positive patients. The sensitivity and NPV of the DETECT algorithm decreased to 97.1% and 92.3%, with one ILD-associated PH (WHO group 3 PH) patient missed. ESC/ERS guidelines did not miss any extra patients except for the PAH patient as noted above. For screening for all types of PH patients, the three models performed equally in sensitivity and NPV. The DETECT algorithm missed one WHO group 3 patient, the ASIG algorithm missed one WHO group 2 patient, and the ESC/ERS guidelines missed one WHO group 1 patient. The specificities of these three models for screening for precapillary PH and PH were very similar to those seen in screening for PAH, and the PPVs increased slightly. The ASIG algorithm still performed the best with respect to specificity and PPV (Table 4).

**Table 4:** **Comparison of the performance of DETECT versus ESC/ERS versus ASIG screening models for precapillary pulmonary hypertension and pulmonary hypertension in patients with systemic sclerosis**

	**Precapillary PH** ^**a**^ **versus non-PH**			**PH** ^**b**^ **versus non-PH**		
	**DETECT**	**ESC/ERS**	**ASIG**	**DETECT**	**ESC/ERS**	**ASIG**
	**n = 69**	**n = 66**	**n = 42**	**n = 73**	**n = 70**	**n = 46**
**Positive** ^**c**^	56 (81.2)	57 (86.4)	30 (71.4)	60 (82.2)	61 (87.1)	33 (71.7)
**Negative** ^**c**^	13 (18.8)	9 (13.6)	12 (28.6)	13 (17.8)	9 (12.9)	13 (28.3)
**True PAH On RHC** ^**d**^	35 (50.7)	35 (53.0)	20 (47.6)	39 (53.4)	39 (55.7)	24 (52.2)
**Sensitivity**	97.1%	97.1%	100%	97.4%	97.4%	95.8%
**(95% CI)**	(85.1-99.9)	(85.1-99.9)	(83.2-100)	(86.5-99.9)	(86.5-99.9)	(78.9-99.9)
**Specificity**	35.3%	25.8%	54.5%	35.3%	25.8%	54.5%
**(95% CI)**	(19.7-53.5)	(11.9-44.6)	(32.2-75.6)	(19.7-53.5)	(11.9-44.6)	(32.2-75.6)
**PPV**	60.7%	59.6%	66.7%	63.3%	62.3%	69.7%
**(95% CI)**	(46.8-73.5)	(45.8-72.4)	(47.2-82.7)	(49.9-75.4)	(49.0-74.4)	(51.3-84.4)
**NPV**	92.3%	88.9%	100%	92.3%	88.9%	92.3%
**(95% CI)**	(64.0-99.8)	(51.8-99.7)	(73.5-100)	(64.0-99.8)	(51.8-99.7)	(64.0-99.8)

^a^Precapillary pulmonary hypertension (PH) means World Health Organization (WHO) group 1 (PAH) and 3 (lung disease/hypoxia-associated) PH. ^b^PH means WHO 1, 2 (left heart disease-associated), and 3 PH. ^c^Positive or negative number screened by each of the algorithms. Values are presented as number (percentage). ^d^True PAH number confirmed by right heart catheterization (RHC). Values are presented as number (percentage). ASIG, Australian Scleroderma Interest Group; CI, confidence interval; ESC/ERS, European Society of Cardiology/European Respiratory Society; NPV, negative predictive value; PAH, pulmonary arterial hypertension; PPV, positive predictive value.

## Discussion

The DETECT algorithm is a novel evidence-based screening model for PAH in patients with SSc and was developed from a worldwide multi-center cross-sectional study. This algorithm was verified in the derivation cohort to have high sensitivity and NPV [14], which are thought to be the most important properties for evaluating a screening algorithm. The present study is the first to evaluate the performance of the DETECT algorithm among patients who were not included in the derivation study. In our study, DETECT performed well, with sensitivity and NPV of 100%. No PAH patients were missed by this algorithm in this cohort. Compared with ESC/ERS guidelines, the DETECT algorithm involves more variables and does not rely just on symptoms and TRV; therefore, it can be used even in patients with undetectable TRV. The eight variables included in the final DETECT algorithm were derived from univariable and multivariable statistical analyses; some of these variables have previously been verified as predictive factors for the presence of SSc-PAH, such as FVC/DLCO [8-10], NT-proBNP [11,12], telangiectasia [20], ACA [21,22], serum urate [23,24], and TRV [2]. In our univariable comparison between PAH and non-PH groups, significant difference were found in seven of the eight variables. Right axis deviation on ECG was not found to be different between these two groups. These results further verify the plausibility of these selected variables. The variables in the DETECT algorithm are easy to measure in clinical practice. The nomogram in the original DETECT article is easy to use for calculating the risk points, but the process is time-consuming. The recently released calculator for this algorithm is easier and quicker to use than the nomogram and can be accessed online [17].

It is important to note that the entry criteria for the DETECT study were a DLCO of less than 60% and SSc disease duration of more than 3 years. However, in clinical practice, these restrictions may miss some PAH patients who have either early SSc or a preserved DLCO. Therefore, to assess the performance of DETECT in a less selective group of patients, we did not apply these entry criteria in the present study. In fact, in our study, 6.5% of PAH patients (n = 4) had DLCO greater than 60% and 8.2% of PAH patients (n = 5) had disease duration of less than 3 years. If we had applied these entry criteria of the original DETECT study, we would have missed these patients. Hence, our study has shown that the DETECT algorithm may be reliably applied to patients with DLCO of more than 60% and disease duration of less than 3 years.

The ASIG algorithm was published shortly before DETECT, and the higher specificity (54.5%), NPV (92.3%), and PPV (61.5%) compared with the ESC/ERS guidelines have been verified by a recent validation study [13]. In the present study, we compared the performance of ASIG and DETECT and found that, compared with the DETECT algorithm, the ASIG algorithm performed equally well with respect to sensitivity and NPV. The ASIG algorithm reduced the referral rate for RHC by 12% compared with the DETECT algorithm but did not increase the number of ‘missed’ cases. Thus, it may be possible to rationalize the use of this invasive procedure. In the ASIG algorithm, only two tests are required, which makes clinical assessment easier and more economical.

Compared with the other two algorithms, ESC/ERS guidelines had lower sensitivity and NPV. One PAH patient was missed by the algorithm because of very mild TRV and normal right heart size. However, this patient had some indicators of PAH (for example, an extremely low DLCO percentage predicted and high FVC/DLCO ratio), which may be the reasons why this patient was not missed by the DETECT and ASIG algorithms. It must also be noted that the ESC/ERS guidelines could not be applied to three patients in whom there was no TR jet on TTE.

At present, in clinical practice, the ESC/ERS guidelines rely mainly on TTE but are still the main screening tool for PAH. Furthermore, doctors are familiar with TTE and often judge the likelihood of PAH through measurement of TRV only. However, TRV cannot be obtained in 20% to 39% of patients [6,7]. In our cohort, if TRV were used as a single assessment tool, 4% of PAH patients would be missed when using a PAH suspicion threshold of at least 2.8 m/s, and 48% would be missed when using a threshold of more than 3.4 m/s.

In clinical practice, in addition to WHO group 1 PAH, WHO groups 2 and 3 PH occur in patients with SSc [25,26]. According to data from the North American PHAROS (Pulmonary Hypertension Assessment and Recognition of Outcomes in Scleroderma) study, WHO groups 2 and 3 PH account for 10% and 21%, respectively, of all types of PH in patients with SSc [26]. The symptoms in patients with WHO groups 2 and 3 PH are similar to those of patients with WHO group 1 PAH, and the prognosis of these patients may be even worse than that of the group 1 patients. Two studies from UK and France both showed the prognosis of SSc patients with respiratory disease-associated PH (WHO group 3) was significantly worse than the prognosis of patients with isolated SSc-PAH (WHO group 1) [21,27]. This suggests that it may be important to identify all types of PH as early as possible. For this reason, although the DETECT and ASIG algorithms were derived in patients with WHO group 1 PAH, their performance in screening for WHO groups 2 and 3 PH was also assessed in the present study. The DETECT algorithm missed one WHO group 3 PH patient, and the ASIG algorithm missed one WHO group 2 PH, but generally both algorithms were still effective in detecting these types of PH. Of course, the small case number in these two groups limits the interpretation of the findings. According to the original article, if applied to the total SSc PH population, the DETECT algorithm could miss as many as 19% of WHO group 2 PH patients and 37% of WHO group 3 PH patients [14].

Despite the limitations of the three screening algorithms we evaluated, they each performed reasonably well in detecting SSc-PAH, echoing the sentiments of many experts that any reasonable screening algorithm applied for the early detection of SSc-PAH is better than usual care. That said, accuracy, accessibility, and economic considerations are likely to influence the choice of algorithm for SSc-PAH screening.

This study has some limitations. Firstly, the patients in this study were a population enriched for PAH, although this was also the case for the DETECT and ASIG derivation studies. Therefore, the prevalence of PAH was significantly higher than the expected prevalence of 10% in an unselected SSc population, as reported in the literature [19]. Our alternate case scenario analysis showed that in the case where the prevalence was set at 10%, the PPVs decreased significantly to lower than 20%, although the NPVs were still very high. Owing to ethical considerations, it is difficult to perform an RHC in all patients with SSc, especially those who are at very low risk of having PAH.

Secondly, as this was a retrospective study, the ability to avoid missed data is limited, so some patients with more than one missing variable were excluded from the analyses. Another limitation is the small case numbers, especially for validating the performance of the ASIG algorithm, as patients involved in the original derivation study for the ASIG algorithm were excluded. Furthermore, although the patients to whom the three screening algorithms were applied in the current study were not included in the original ASIG algorithm derivation study, they were still recruited from the same source. This may have enhanced the performance of the ASIG algorithm in this setting. Accordingly, the findings should be confirmed in a larger external group of patients. The impact of concomitant immunosuppressive therapy on results of screening with each of the algorithms also requires evaluation in future studies. Finally, patients with severe ILD were excluded from this study. The performance of screening algorithms such as DETECT and ASIG in the setting of coexistent ILD merits further evaluation.

## Conclusions

In summary, the DETECT and ASIG algorithms out-perform the ESC/ERS guidelines, with a high sensitivity, which is the most important feature for a screening algorithm, reducing or eliminating missed diagnoses. The ESC/ERS guidelines have limitations in patients with undetected or mild TR jet. The specificity of all of the screening models is low, as may be expected, but the ASIG algorithm performed better with respect to specificity and reduced the number of referrals for RHC. Ultimately, it is likely that the choice of SSc-PAH screening algorithm will also depend on cost and ease of application.

## Additional file

Additional file 1:
**Details of the ‘alternate case scenario analysis’.** Description: Alternate case scenario analysis assuming pulmonary arterial hypertension (PAH) prevalence of 10%.

## Abbreviations

6MWD: 6-minute walk distance
ACA: Anti-centromere antibody
ASCS: Australian Scleroderma Cohort Study
ASIG: Australian Scleroderma Interest Group
DLCO: Diffusing capacity for carbon monoxide
ECG: Electrocardiogram
ESC/ERS: European Society of Cardiology/European Respiratory Society
FVC: Forced vital capacity
FVC/DLCO: Forced vital capacity (percentage predicted)/diffusing capacity for carbon monoxide (percentage predictive)
HRCT: High-resolution computed tomography
ILD: Interstitial lung disease
mPAP: Mean pulmonary artery pressure
NPV: Negative predictive value
NT-proBNP: N-terminal pro-B type natriuretic peptide
PAH: Pulmonary arterial hypertension
PCWP: Pulmonary capillary wedge pressure
PFT: Pulmonary function test
PPV: Positive predictive value
RA: Right atrium
RHC: Right heart catheterization
sPAP: Systolic pulmonary artery
SSc: Systemic sclerosis
SSc-PAH: Systemic sclerosis-associated pulmonary arterial hypertension
TR: Tricuspid regurgitant
TRV: Tricuspid regurgitant jet velocity
TTE: Transthoracic echocardiography
V/Q: Ventilation-perfusion
WHO: World Health Organization
WHO FC: World Health Organization functional class
