# Supplementary material for: A comparison of the predictive accuracy of three screening models for pulmonary arterial hypertension in systemic sclerosis
Source: Arthritis Res Ther. 2015 Jan 18;17(1):7. doi: 10.1186/s13075-015-0517-5 (PMC4332896; doi:10.1186/s13075-015-0517-5)
Supplement: Additional file 1: — Details of the ‘alternate case scenario analysis’. Description: Alternate case scenario analysis assuming pulmonary arterial hypertension (PAH) prevalence of 10%. [file 13075_2015_517_MOESM1_ESM.pdf]

Additional file

Details of the "alternate case scenario analysis"

An alternate case scenario analysis was performed, setting the prevalence of PAH to the commonly accepted 10%, using the previously determined point estimates of sensitivity and specificity. The equations below were used to calculate the adjusted positive predictive value (PPV) and negative predictive value (NPV):

$$PPV = \frac{(sensitivity)*(prevalence)}{(sensitivity)*(prevalence) + (1-specificity)*(1-prevalence)}$$

$$NPV = \frac{(specificity)*(1-prevalence)}{(specificity)*(1-prevalence) + (1-sensitivity)*(prevalence)}$$

1. DETECT screening algorithm

Using the equations above, and assuming an SSc-PAH prevalence=10%, sensitivity=100% and specificity=35.3%, the 'adjusted' PPV and NPV for SSc-PAH using the DETECT screening algorithm were 14.7% and 100%, respectively.

Supplementary Table 1 - Two-by-two tables for the DETECT screening algorithm assuming an SSc-PAH prevalence of 10%.

		SSc-PAH		Total
		Yes	No	
DETECT screening algorithm	Positive	6	35	41
	Negative	0	20	20
Total		6	55	61

2. ASIG screening algorithm

Assuming an SSc-PAH prevalence=10%, sensitivity=100% and specificity=54.5%, the 'adjusted' PPV and NPV for SSc-PAH using the ASIG screening algorithm were 19.6% and 100%, respectively.

Supplementary Table 2 - Two-by-two tables for the ASIG screening algorithm assuming an SSc-PAH prevalence of 10%.

		SSc-PAH		Total
		Yes	No	
ASIG screening algorithm	Positive	4	16	20
	Negative	0	17	17
Total		4	33	37

3. ESC/ERS screening guidelines

Assuming an SSc-PAH prevalence =10%, sensitivity = 96.3%, specificity=32.3%, the 'adjusted' PPV and NPV for SSc-PAH using the ESC/ERS screening model were 13.6% and 98.7%, respectively.

Supplementary Table 3 - Two-by-two tables for theESC/ERS screening model assuming an SSc-PAH prevalence of 10%.

		SSc-PAH		
		Yes	No	Total
ESC/ERS screening guidelines	Positive	6	38	44
	Negative	0	14	14
Total		6	52	58
